# IQGAP1-Dependent Signaling Pathway Regulates Endothelial Cell Proliferation and Angiogenesis

**DOI:** 10.1371/journal.pone.0003848

**Published:** 2008-12-03

**Authors:** Rosana D. Meyer, David B. Sacks, Nader Rahimi

**Affiliations:** 1 Departments of Pathology and Ophthalmology, School of Medicine, Boston University, Boston, Massachusetts, United States of America; 2 Department of Pathology, Brigham and Women's Hospital and Harvard Medical School, Boston, Massachusetts, United States of America; University of Cambridge, United Kingdom

## Abstract

**Background:**

Vascular endothelial growth factor receptor-2 (VEGFR-2) signaling is an obligate requirement for normal development and pathological angiogenesis such as cancer and age-related macular degeneration. Although autophosphorylation of tyrosine 1173 (Y1173) of VEGFR-2 is considered a focal point for its angiogenic signal relay, however, the mechanism of phosphorylation of Y1173, signaling proteins that are recruited to this residue and their role in angiogenesis is not fully understood.

**Methodology/Principal Findings:**

In this study we demonstrate that c-Src kinase directly through its Src homology 2 (SH2) domain and indirectly via c-Cbl binds to phospho-Y1057 of VEGFR-2. Activation of c-Src kinase by a positive feedback mechanism phosphorylates VEGFR-2 at multi-docking site, Y1173. c-Src also catalyzes tyrosine phosphorylation of IQGAP1 and acts as an adaptor to bridge IQGAP1 to VEGFR-2. In turn, IQGAP1 activates b-Raf and mediates proliferation of endothelial cells. Silencing expression of IQGAP1 and b-Raf revealed that their activity is essential for VEGF to stimulate angiogenesis in an *in vivo* angiogenesis model of chicken chorioallantoic membrane (CAM).

**Conclusions/Significance:**

Angiogenesis contributes to the pathology of numerous human diseases ranging from cancer to age-related macular degeneration. Determining molecular mechanism of tyrosine phosphorylation of VEGFR-2 and identification of molecules that are relaying its angiogenic signaling may identify novel targets for therapeutic intervention against angiogenesis-associated diseases. Our study shows that recruitment and activation of c-Src by VEGFR-2 plays a pivotal role in relaying angiogenic signaling of VEGFR-2; it phosphorylates VEGFR-2 at Y1173, facilitates association and activation of IQGAP1 and other signaling proteins to VEGFR-2. IQGAP1-dependent signaling, in part, is critically required for endothelial cell proliferation, a key step in angiogenesis. Thus, Y1057 of VEGFR-2 serves to regulate VEGFR-2 function in a combinatorial manner by supporting both diversity of recruitment of angiogenic signaling proteins to VEGFR-2, and its ability to promote angiogenesis.

## Introduction

Activation of vascular endothelial growth factor receptor-2 (VEGFR-2 also called FLK-1/KDR) plays a pivotal role in mediating growth of blood vessels, angiogenesis [1]. VEGF stimulation of VEGFR-2 provokes pleiotropic responses in endothelial cells including endothelial cell growth, survival, differentiation, migration, and tube formation [2]. Autophosphorylation of tyrosine 1173 (Y1173) of VEGFR-2 is a focal point for activation of angiogenic signal relay of VEGFR-2, as it has emerged as a multi-functional docking site involved in the recruitment of multiple signaling proteins including the p85 regulatory subunit of phosphatidylinositol 3-kinase (p85/PI3-K) [3], [4], phospholipase Cγ1 (PLCγ1) [5], [6], Shb [7], and the Shc-related adaptor protein, Sck [8]–[10] and the transmission of a range of biological signals to coordinate endothelial cell function and angiogenesis. The critical and direct role of Y1173 in enabling VEGFR-2 to promote angiogenesis was recently revealed by a gene targeting study where mice homozygous for the mutant *VEGFR-2* ^Y1173F^ knock-in allele died between E8.5 and E9.5 without any organized blood vessels [11] similar to the VEGFR-2 null mice [12]. More recent studies indicate that in addition to Y1173, the kinase domain tyrosine, Y1057 also takes part in VEGFR-2 function and is involved in VEGFR-2-mediated cell proliferation [13]. Y1057 along with Y1173 also is engaged in the recruitment and activation of the ubiquitin E3 ligase, c-Cbl. Phospho-Y1057 contributes to the recruitment of c-Cbl to VEGFR-2 by directly associating with c-Cbl, while phospho-Y1173 indirectly via PLCγ1 participates in its recruitment to VEGFR-2 [14], resulting in the ubiquitination of PLCγ1 and inhibition of angiogenesis *in vitro* [14].

The recent study indicates that IQGAP1 associates with VEGFR-2 and its activity is required for proliferation of endothelial cells *in vitro* [26]. It remains, however, unclear how IQGAP1 interacts with VEGFR-2 since it lacks phospho-tyrosine binding domains such as SH2 and PTB and how its activity is regulated by VEGFR-2. IQGAP1 contains multiple protein-interaction domains including calponin homology domain (CHD), poly-proline-binding domain (WW), calmodulin-binding domain (IQ) and rasGTPase-activating protein (GAP)-related domain (GRD) and tyrosine and serine/threonine phosphorylation sites [33] and is involved in multiple cellular functions including calcium/calmodulin signaling, MAPK signaling and regulation of cytoskeletal structure, cell adhesion and cell motility [27], [31], [33].

In this study, we uncovered a surprising correlation between phosphorylation of Y1057 and Y1173 and that phosphorylation of Y1057 plays a multitasking role in mediating VEGFR-2-directed angiogenic signaling events in endothelial cells. Phospho-Y1057 recruits c-Src kinase to VEGFR-2, and in part catalyzes phosphorylation of Y1173 via Src kinases. c-Src also bridges IQGAP1 to VEGFR-2 and directly phosphorylates IQGAP1 and promotes endothelial cell proliferation, a key step in angiogenesis.

## Results

### Identification of tyrosine 1057 of VEGFR-2 as a binding site for Src kinases

**Figure 1:**
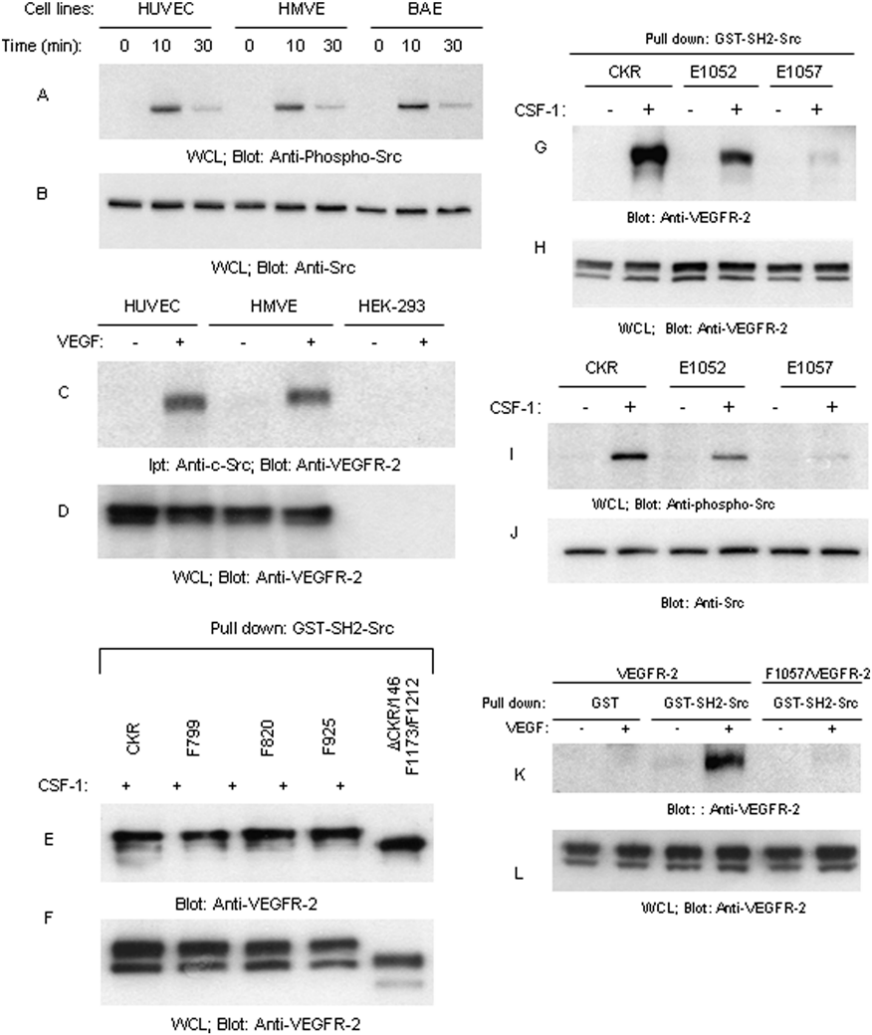
Tyrosine 1057 mediates recruitment of c-Src to and its activation by VEGFR-2. HUVEC, HMVE and BAE cells were stimulated with VEGF-A (100 ηg/ml) for indicated times. Whole cell lysates (WCL) were immunoblotted with an anti-phospho-Src (pY416) antibody (A) or an anti-Src antibody (B). HUVEC, HMVE and HEK-293 cells were either unstimulated (−) or stimulated with VEGF (+) for 10 minutes, lysed, immunoprecipitated with c-Src antibody and immunoblotted with an anti-VEGFR-2 antibody (C). Whole cell lysates (WCL) from the same group was immunoblotted with an anti-VEGFR-2 as a control (D). PAE cells expressing wild type chimeric VEGFR-2 (CKR) and tyrosine mutant CKRs, F799/CKR, F820/CKR, F925/CKR and carboxyl terminus deleted CKR coupled with mutation of Y1173 and Y1212 denoted as ΔCKR/F1173/F1212 was stimulated with CSF-1 for 10 minutes. Cells were lysed, and whole cell lysates were incubated with purified GST-SH2-Src protein. The GST-SH2-Src bound proteins were subjected to Western blot analysis using an anti-VEGFR-2 antibody (E). Whole cell lysates from the same groups was blotted with an anti-VEGFR-2 antibody (F). PAE cells expressing CKR, E1052/CKR, and E1057/CKR were either unstimulated or stimulated with CSF-1 for 10 minutes and cells were lysed and cell lysates was subjected to GST-SH2-Src pull-down assay (G). An immunoblot of whole cell lysates also were probed with an anti-VEGFR-2 antibody (F,H), an anti-phospho-Src (pY416) antibody (I) or an anti-Src antibody (J). PAE cells expressing VEGFR-2 or F1057/VEGFR-2 were prepared and subjected to pull-down analysis as panel E (K) or whole cell lysates was blotted with an anti-VEGFR-2 antibody (L). The results shown in A–L are representative of three separate experiments.

Although stimulation of VEGFR-2 in endothelial cells is known to promote activation of Src kinases [15] and Src activation is linked to endothelial cell permeability, survival and angiogenesis [16], [17], the nature of its association with VEGFR-2, in particular, the putative phospho-tyrosine residue on VEGFR-2 involved in its recruitment is not. Our survey of several primary endothelial cells including, HUVEC, HMVE and BAEC cells showed that c-Src is ubiquitously tyrosine phosphorylated in response to VEGF stimulation (Figure 1A) and associates with VEGFR-2 in a ligand-dependent manner in these primary endothelial cells (Figure 1C). Cell lysates obtained from HEK-293 (human embryonic kidney-293) cells which do not express VEGFR-2 was negative for VEGFR-2 and was used as a negative control (Figure 1C). The cytoplasmic region of VEGFR-2 contains multiple tyrosine phosphorylation sites [18], albeit none of these tyrosine autophosphorylation sites shows obvious sequence similarities to the preferred Src SH2 recognition motif, pYEEI [19]. To analyze association of c-Src with VEGFR-2 we utilized the previously characterized VEGFR-2 chimera [20] herein called CKR which is created by replacing the extracellular domain of VEGFR-2 with that of human Colony stimulating factor-1 receptor (CSF1R) and expressed in PAE (porcine aortic endothelial) cells as an experimental system. In our previous studies we have extensively characterized CKR with VEGFR-2 in a comparative manner. CKR in term of its ability undergo down-regulation, activation, recruitment of signaling proteins and to stimulate cellular responses is identical to VEGFR-2 [3], [14], [20], [21] and data presented in this manuscript. We used this strategy to avoid cross-talk between VEGFR-2 and other VEGF receptors, in particular, VEGFR-1. Our initial observation demonstrated that mutation of tyrosines 799, 820, 925 to phenylalanine (F) and deletion of C-terminus of VEGFR-2 coupled with mutation of tyrosines 1173 and 1212 has no effect on the ability of VEGFR-2 to associate with the SH2 domain of c-Src (Figure 1E). In contrast, mutation of Y1052 to glutamic acid (E) and particularly Y1057, located in the kinase domain, severely reduced the binding of VEGFR-2 with SH2 domain of c-Src (Figure 1G). GST alone does not associate with CKR (Figure S3). Consistent with its reduced binding capability to E1052, the result showed that E1052 only partially reduces the ability of VEGFR-2 to phosphorylate c-Src (Figure 1I). In contrast, the potential of mutant Y1057 (E1057/CKR) to stimulate Src tyrosine phosphorylation was severely compromised and was near to undetectable (Figure 1I). Since both Y1052 and Y1057 are located in the kinase domain we replaced these residues to glutamic acid (E) rather than phenylalanine (F). This type of mutations has been suggested to be useful for characterization of sensitive tyrosine sites on the RTKs (receptor tyrosine kinases) when their mutation to phenylalanine interferes with receptor activation [15]. However, unlike these RTKs, single mutation of Y1052 and Y1057 either to E or F did not interfere with activation of VEGFR-2 in the context of CKR (Figures S1 and S2). Furthermore, to make sure that our observation in CKR also is true in the context of non-chimeric VEGFR-2, we mutated Y1057 to F in the full length VEGFR-2, expressed in PAE cells and similarly analyzed for its ability to bind SH2 domain of c-Src. The result showed that VEGFR-2 associates with SH2-Src in VEGF-dependent manner and mutation of Y1057 inhibited association of VEGFR-2 with c-Src (Figure 1K). Altogether, the data show that Y1057 of VEGFR-2 is a major c-Src binding site on VEGFR-2 and its presence is critically required for VEGFR-2 to activate c-Src.

### c-Cbl associates with c-Src and participates in the interaction of c-Src with VEGFR-2

**Figure 2:**
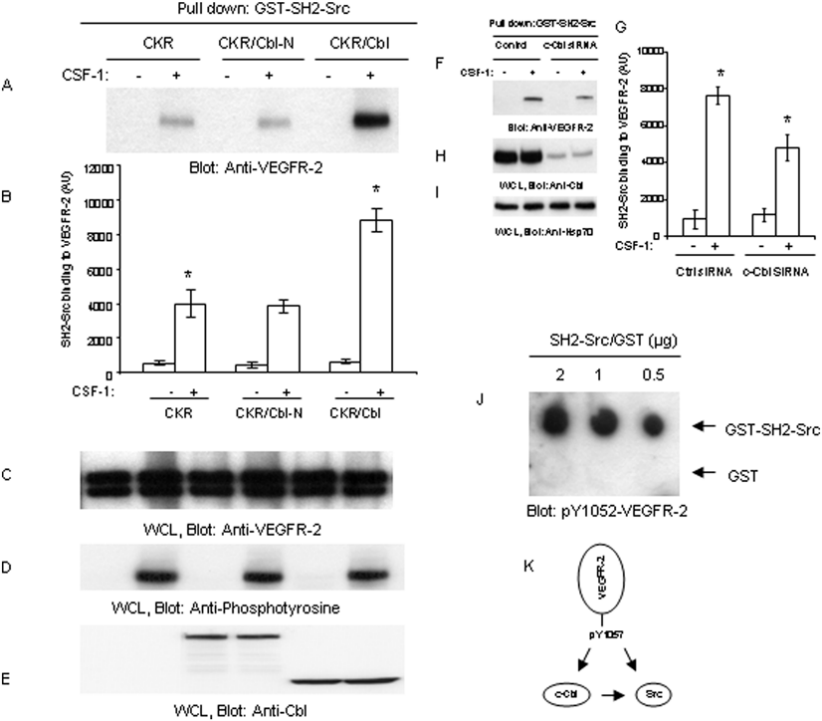
c-Cbl-dependent and independent association of c-Src with VEGFR-2. Serum-starved PAE cells expressing chimeric VEGFR-2 (CKR) or co-expressing CKR with c-Cbl and CKR with Cbl-N were either unstimulated (−) or stimulated with CSF-1 (+) for 10 minutes. Cells were lysed and subjected to an in vitro pull-down assay using purified GST-SH2-Src protein (A). Quantification of blots of SH2-Src binding to VEGFR-2 of three separate experiments is shown. Each bar represents the mean ±SD of triplicate experiments. *P<0.01 (B). A parallel immunoblot of whole cell lysates (WCL) was probed with an anti-VEGFR-2 antibody (C), with an anti-phosphotyrosine antibody (D) and with an anti-c-Cbl antibody (E). PAE cells co-expressing chimeric VEGFR-2 (CKR) with a control siRNA or Cbl-siRNA were stimulated with CSF-1 for 10 minutes and cells were lysed and prepared for in vitro pull-down assay as panel A (F). A parallel immunoblot of whole cell lysates were probed with an anti-c-Cbl antibody for confirmation of c-Cbl knockdown (H) and anti-Hsp70 antibody as a control for protein loading (I). To detect a direct interaction between the SH2 domain of c-Src and Y1057 of VEGFR-2, the indicated quantities of purified recombinant GST-SH2-Src (top) and GST control (bottom row) were dot blotted as described in the Materials and Methods and detected by an anti-pY1057 VEGFR-2 antibody (J). The graph represents the average binding of SH2-Src to VEGFR-2 in the absence or presence of c-Cbl-siRNA (±SD) of three separate experiments. Quantification of blots of SH2-Src binding to VEGFR-2 of three separate experiments is shown. *P<0.01 (G). All the experiments repeated at least three times. A summary of the proposed interaction of c-Src with VEGFR-2 is shown (K).

A recent study indicates that tyrosine 1057 of VEGFR-2 also serves as a binding site for c-Cbl by directly interacting with its tyrosine kinase binding (TKB) domain [14], raising an interesting possibility as to whether c-Cbl and c-Src competitively bind to Y1057 or c-Cbl acts to bridge c-Src to VEGFR-2 since c-Cbl is known to interact with c-Src via its SH3 domain [22]. To test these two distinct possibilities, we used PAE cells co-expressing CKR either with wild type c-Cbl or Cbl-N, where the TKB domain is deleted [14] and analyzed the ability of GST-SH2 domain of c-Src to interact with VEGFR-2. Over-expression of wild type c-Cbl enhanced association of SH2 domain of c-Src with VEGFR-2, where disabling the binding of c-Cbl with VEGFR-2 had no effect on the binding of SH2-Src to VEGFR-2 (Figure 2A). Quantification of the blots (three independent experiments) showed that indeed interaction of VEGFR-2 with GST-SH2-Src is more than doubled when c-Cbl is over-expressed, where as the deletion of TKB domain almost entirely is inhibited this effect of c-Cbl (Figure 2B). Over-expression of either c-Cbl or c-Cbl-N had no effect on the protein levels of CKR or on its tyrosine phosphorylation (Figure 2C, 2D). Expression of c-Cbl and c-Cbl-N also is shown (Figure 2E). Since up-regulation of c-Cbl by over-expression enhanced association of c-Src with VEGFR-2, we further tested whether silencing expression of c-Cbl by siRNA would reduce interaction of SH2-Src with VEGFR-2. As shown, silencing the expression of c-Cbl in PAE cells reduced the binding of GST-SH2 Src with VEGFR-2 (Figure 2F). Quantification of the blots (three independent experiments) is also shown which further support the hypothesis that endogenous c-Cbl plays a role in bridging c-Src to VEGFR-2 (Figure 2G). The silencing effect of c-Cbl-siRNA on the c-Cbl protein level also is shown (Figure 2H). It showed be noted that c-Cbl is highly expressed in these cells and siRNA only partially reduced its expression (Figure 2H). Altogether, the data show that c-Cbl, in part, facilitates association of Src with VEGFR-2. To firmly establish a direct binding of SH2 domain of c-Src with phospho-Y1057 of VEGFR-2 we synthesized a phosphorylated peptide corresponding to Y1057 and tested its binding ability to GST-SH2 domain of c-Src in an *in vitro* dot blot assay. The result shows that SH2 domain of Src also directly interacts with phosphorylated Y1057 in a c-Cbl independent manner (Figure 2J), suggesting that the interaction of c-Src with Y1057 of VEGFR-2 is established by a direct binding involving its SH2 domain and an indirect interaction involving c-Cbl. Figure 2K summarizes our observation regarding interaction of c-Src with VEGFR-2 and role of c-Cbl in this process.

### Tyrosine 1173 of VEGFR-2 is phosphorylated by Src kinases

**Figure 3:**
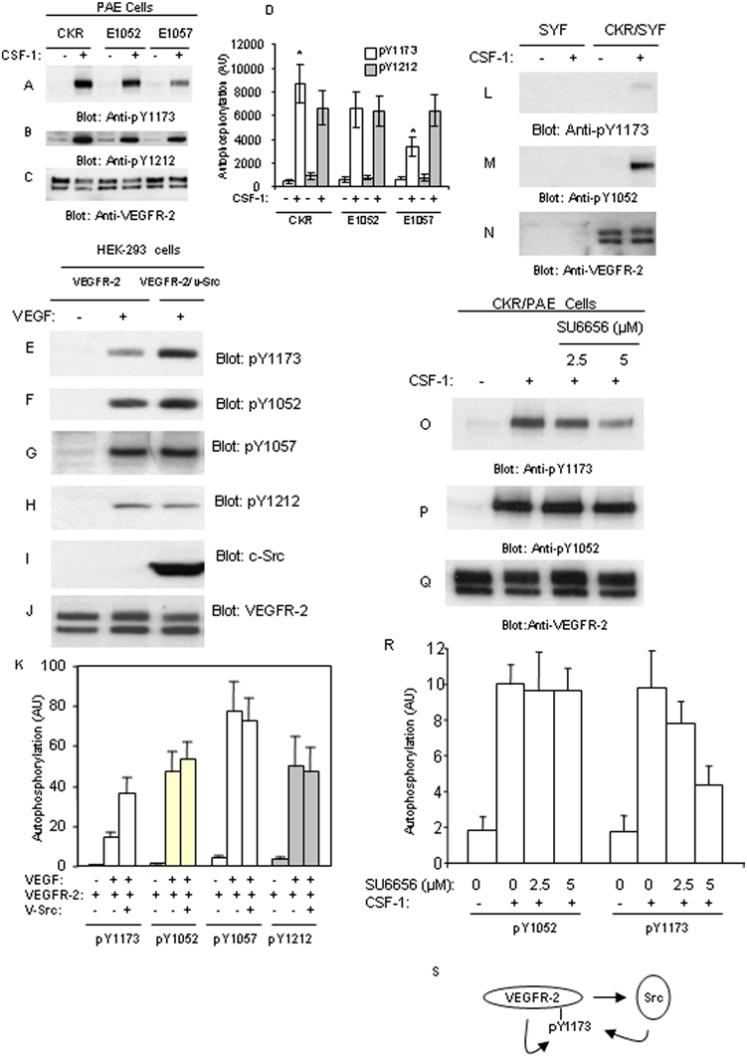
c-Src kinase activity is required for maximal phosphorylation of Y1173 of VEGFR-2. Serum-starved PAE cells expressing wild type CKR, E1052/CKR and E1057/CKR were either unstimulated (−) or stimulated with CSF-1 (+) for 10 minutes. Whole cell lysates (WCL) were subjected to Western blot analysis using an anti-phosphoY1173 specific-VEGFR-2 antibody (A), an anti-phosphoY1212 specific-VEGFR-2 antibody (B) or an anti-VEGFR-2 antibody (C). The graph represents the mean phosphorylation of Y1173 and Y1212 of VEGFR-2 (±SD) of three separate experiments. *P<0.01 (D). HEK-293 cells transiently expressing VEGFR-2 alone or co-expressing v-Src were stimulated with VEGF and immunoblotted with anti-pY1173 (E), anti-pY1052 (F), anti-pY1057 (G), anti-pY1212 (H), anti-c-Src (I) and anti-VEGFR-2 (J) antibodies. The blots were also quantified using Image J program (developed by NIH) and represents average tyrosine phosphorylation sites on VEGFR-2 of three separate experiments (±SD) from three separate experiments (K). SYF cells and SYF cells expressing chimeric VEGFR-2 (CKR) were either unstimulated (−) or stimulated with CSF-1 (+) for 10 minutes and cells lysates were prepared and immunoblotted with anti-pY1173 (L), anti-pY1052 (M) and anti-VEGFR-2 (N) antibodies. PAE cells expressing CKR were treated with different concentration of SU6656 for 30 minutes and stimulated with CSF-1 for 10 minutes and cell lysates were prepared as panel A and immunoblotted with anti-pY1173 (O), anti-pY1052 (P) and anti-VEGFR-2 (Q) antibodies. Phosphorylation of Y1173 and Y1052 in response to SU6656 treatment was quantified as panel D and is presented as the mean (±SD) of three separate independent experiments (R). A summary of the proposed model of phosphorylation Y1173 of VEGFR-2 by c-Src and VEGFR-2 itself is shown (S).

Tyrosine 1173 functions as a multitasking residue on VEGFR-2 [3]–[7], [10] and is a bona fide mediator of angiogenic signaling of VEGFR-2 *in vivo* [11]. Hence we investigated a possible crosstalk between the kinase domain tyrosines (i.e., Y1052 and Y1057) and Y1173 and the potential role of Src kinases in this crosstalk. For this aim, we tested phosphorylation of Y1173 in the background of E1052/CKR and E1057/CKR expressed in PAE cells. The result showed that mutation of 1057 markedly reduced ligand-dependent phosphorylation of Y1173 as detected by an anti-phospho-Y1173 specific antibody (Figure 3A). Mutation of Y1052 only slightly reduced phosphorylation of Y1173, suggesting that Y1057 but not Y1052, is specifically involved in modulation of phosphorylation of Y1173 (Figure 3A). However, mutation of either Y1052 or Y1057 had no major effect on the phosphorylation Y1212 (Figure 3B). Quantification of phosphorylation of Y1173 and Y1212 obtained from three separate independent experiments also is shown (Figure 3D). Since Src kinase binds to Y1057 of VEGFR-2, we tested potential role of Src in the phosphorylation of Y1173. Our analysis showed that co-expression of v-Src with non-chimeric wild-type VEGFR-2 in a transient transfection of HEK-293 cells also increases phosphorylation of Y1173 (Figure 3D), where phosphorylation of Y1052, Y1057 and Y1212 were not effected (Figure 3F–H). The quantification of phosphorylation of Y1173, Y1052, Y1057 and Y1212 also is shown (Figure 3K). Of note, co-expression of v-Src with E1057 mutant VEGFR-2 also rescued the reduced phosphorylation of Y1173 further suggesting that c-Src is involved in catalyzing phosphorylation of Y1173 of VEGFR-2 (data not shown).

To further link Src kinase activity to phosphorylation of Y1173 of VEGFR-2, we tested the phosphorylation of Y1173 of VEGFR-2 in the triple knockout SYF (c-Src, c-Yes and c-Fyn) cells [24]. The data showed that stimulation of SYF cells ectopically expressing chimeric VEGFR-2 (CKR) with ligand increases phosphorylation of Y1052. In contrast, phosphorylation of VEGFR-2 at Y1173 was almost undetectable (Figure 3L and 3M). Only a long exposure of the film detected a weak phosphorylation of Y1173 (data not shown), suggesting that in addition to Src kinases, VEGFR-2 itself also catalyzes phosphorylation of Y1173 of VEGFR-2. Since in the recent years various Src inhibitors were used for therapeutic purposes in cancer and anti-angiogenesis treatments [25], we tested the effect of Src-specific inhibitor, SU6656 to inhibit phosphorylation of VEGFR-2 at Y1173. The result showed that SU6656 selectively inhibits phosphorylation of VEGFR-2 at Y1173 in a dose-dependent manner but had no effect on the phosphorylation of Y1052 (Figure 3O, 3P). The quantification of inhibition of phosphorylation of Y1173 of VEGFR-2 by SU6656 also is shown (Figure 3R). Taken together, the data demonstrate that Src kinases upon activation by VEGFR-2 phosphorylate Y1173 of VEGFR-2 (Figure 3S).

### Role of c-Src in VEGFR-2 mediated endothelial cell tube formation and proliferation

**Figure 4:**
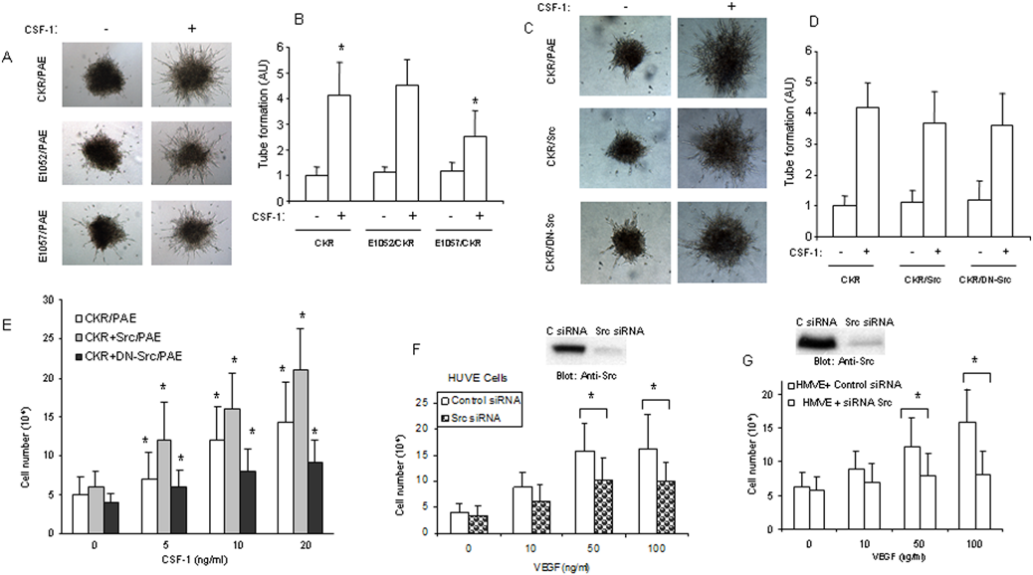
Role of c-Src in VEGFR-2-dependent endothelial cell tubulogenesis and proliferation. PAE cells expressing CKR, E1052/CKR, and E1057/CKR alone or with c-Src and dominant negative c-Src (DN-Src) were prepared as spheroids and subjected to an *in vitro* angiogenesis/tubulogenesis assay as described in the Materials and Methods. The pictures shown are representative spheroids from three independent experiments (A, C). Tubulogenesis was also quantified as the mean of ± SD of four sprouting colonies. *P<0.05 (B, D). Equal number of PAE expressing CKR alone or co-expressing CKR with c-Src, and CKR with DN-Src were subjected to proliferation assay as described in the Materials and Methods and expressed as the mean ± SD of triplicate. *P<0.05 (E). HUVE and HMVE cells treated with either a control siRNA or c-Src-siRNA, after 48 hours cells were stimulated with different concentrations of VEGF and subjected to proliferation as Panel C. *P<0.05 (F, G). The Western blots of cells treated with either control siRNA or c-Src-siRNA also are shown (F, G).

Since activation of Src family kinases is linked to angiogenesis [16], [17] we initially analyzed the ability E1052/CKR and E1057/CKR to stimulate endothelial tubulogenesis *in vitro*. Our analysis showed that mutation of Y1052 had no apparent effect on the ability of CKR to promote tube formation of PAE cells. However, mutation of Y1057 significantly reduced the ability of CKR to stimulate tube formation of PAE cells (Figure 4A). Quantification of tube formation of PAE cells in response to CKR, E1052/CKR and E1057/CKR activation is shown (Figure 4B). To test whether c-Src activation is required for tube formation of PAE cells, we used previously established PAE cells co-expressing CKR with either wild type c-Src or dominantly negative acting kinase inactive Src [15]. The result showed that over-expression of c-Src or dominant negative c-Src in PAE cells do not alter the ability of VEGFR-2 to stimulate tube formation (Figure 4C, 4D), indicating that perhaps c-Src activity is not required for VEGFR-2 to stimulate tube formation of endothelial cells. In contrast, our analysis showed that over-expression of c-Src significantly enhances the ability of VEGFR-2 to stimulate proliferation of PAE cells and over-expression of dominant negative Src (Src kinase-dead) inhibits the VEGFR-2 driven proliferation of PAE cells (Figure 4E). To test the role of c-Src in endothelial cell proliferation further we knocked down c-Src expression in HUVE and HMVE cells by siRNA strategy and tested their VEGF-dependent proliferation. The result showed that silencing the expression of c-Src in endothelial cells significantly inhibits the ability of VEGFR-2 to stimulate proliferation of these primary endothelial cells (Figure 4F, 4G). Interestingly, HMVE cells were more sensitive to silencing of c-Src than HUVE cells since silencing of Src expression almost totally inhibited VEGF-stimulated proliferation (Figure 4G). Taken together, our results identify c-Src as a key component of mitogenic signaling of VEGFR-2 in endothelial cells.

### Identification of IQGAP1 as a novel Src kinase substrate

**Figure 5:**
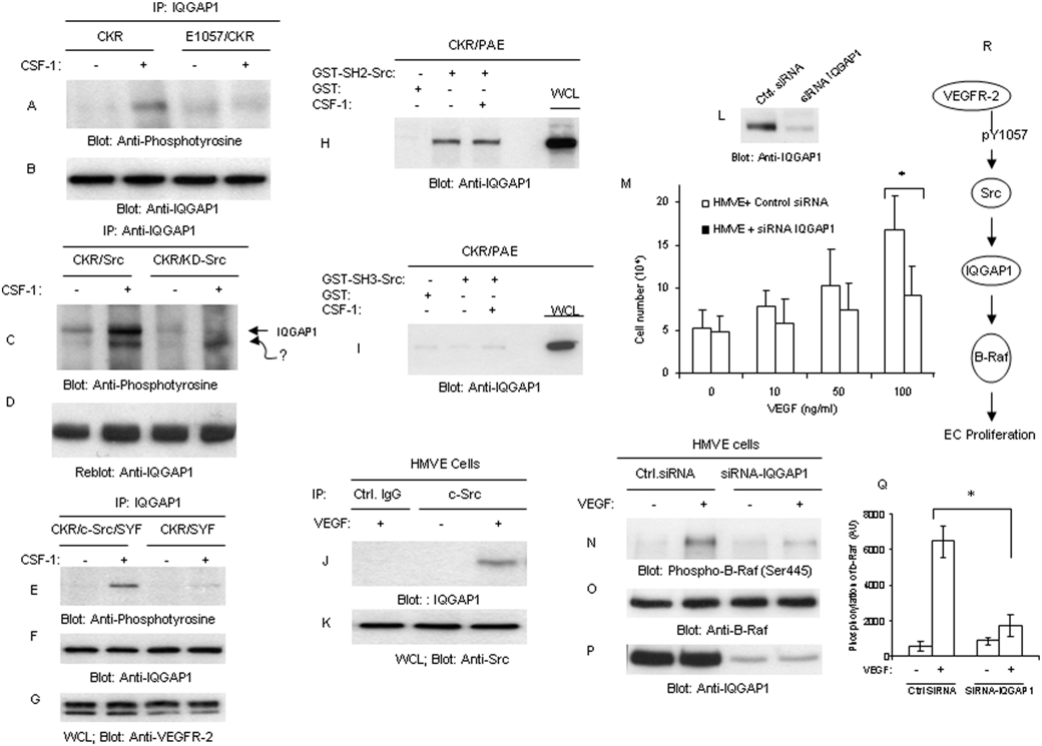
Role of IQGAP1 in VEGFR-2 signaling and endothelial cell Proliferation. Serum-starved PAE cells expressing CKR (chimeric VEGFR-2) and E1057/CKR were either unstimulated (−) or stimulated with CSF-1 (+) for 10 minutes. Whole cell lysates were subjected to immunoprecipitation with IQGAP1 and Western blot analysis using an anti-phosphotyrosine antibody (A) or anti-IQGAP1 antibody (B). PAE cells co-expressing either wild type c-Src or a dominant negative form of c-Src (KD-Src) were stimulated and subjected to Western blot analysis as panel A using an anti-phosphotyrosine antibody (C). The same membrane striped off the antibody and re-blotted with an anti-IQGAP1 antibody for protein levels (D). SYF cells co-expressing CKR with c-Src or expressing CKR alone were prepared as above and blotted with anti-phosphotyrosine (E), anti-IQGAP1 (F), and anti-VEGFR-2 (G). Cell lysates derived from PAE cells expressing CKR were either precipitated with GST, GST-SH2-Src, (H) or GST-SH3-Src (I) and blotted for IQGAP1 using an anti-IQGAP1 antibody. Human primary dermal vascular endothelial (HMVE) cells were stimulated with VEGF for 10 minutes, cells were lysed and immunoprecipitated with either control antibody (Ctrl.IgG) or c-Src antibody and blotted with anti-IQGAP1 (J). Whole cell lysates was blotted with anti-Src antibody (K). HMVE cells expressing either control siRNA (Ctrl.siRNA) or IQGAP1 siRNA were lysed and blotted for IQGAP1 using an anti-IQGAP1 antibody (L). HMVE cells expressing either control siRNA or IQGAP1 siRNA were subjected to proliferation assay as described in Method section. Error bars represent mean ± SD of triplicate samples. *p<0.001 as compared to proliferation of HMVE cells in response to 100 ηg VEGF-A (M). HMVE cells expressing either control siRNA or IQGAP1-siRNA were unstimulated (−) or stimulated (+) with VEGF for 10 minutes and cells were lysed and immunoprecipitated with an anti-b-Raf antibody and blotted with an anti-phospho-Ser445 antibody (N) or with anti- b-Raf antibody (O). Whole cell lysates was blotted for IQGAP1 (P). Also shown is the graph of the mean phosphorylation of b-Raf where the expression of IQGAP1 is silenced by siRNA. It represents (±SD) of three separate experiments. *P<0.01 (Q). Summary of the proposed VEGFR-2-dependent signal relay in endothelial cell proliferation also is shown (R).

IQGAP1 is a scaffold protein and participates in signaling cascades mediated by a diverse group of cell surface receptors [33], including VEGFR-2 [26]. In addition, we recently have identified IQGAP1 as a binding partner of VEGFR-2 by a proteomic approach (our unpublished data), raising the likelihood for the involvement of Y1057 in the recruitment and tyrosine phosphorylation of IQGAP1 by VEGFR-2. Our initial observation showed that IQGAP1 is tyrosine phosphorylated in PAE cells by VEGFR-2 and mutation of Y1057 reduces the ability of VEGFR-2 to stimulate its tyrosine phosphorylation (Figure 5A). IQGAP1 also is tyrosine phosphorylated by other RTKs including, ErbB1/EGFR-1 and PDGFRβ ectopically expressed in PAE cells (our unpublished data), suggesting that IQGAP1 serves as a common substrate for RTKs. In addition, our data show that over-expression of c-Src in PAE cells highly enhanced tyrosine phosphorylation of IQGAP1. In a sharp contrast, over-expression of a dominant negatively acting c-Src inhibited tyrosine phosphorylation of IQGAP1 (Figure 5C).

To further establish role of c-Src in phosphorylation of IQGAP1 we show that in SYF knockout cells where Src family kinases (Yes, Fyn and Src) are absent, IQGAP1 is not tyrosine phosphorylated in response to activation of VEGFR-2, where re-introduction of c-Src rescued phosphorylation of IQGAP1 by VEGFR-2 (Figure 5E). Our further studies showed that in an *in vitro* kinase assay recombinant c-Src protein phosphorylates GST-fusion IQGAP1 (data not shown). Taken together, the data support the hypothesis that IQGAP1 directly associates with c-Src and undergoes c-Src-dependent tyrosine phosphorylation. Hence to further establish as to whether c-Src interacts with IQGAP1 we tested the ability of SH2 and SH3 domains of c-Src to associate with IQGAP1 in an *in vitro* pull-down assay. The result showed that SH2 domain of c-Src interacts with IQGAP1 independent of its tyrosine phosphorylation since SH2 domain of c-Src forms a complex with IQGAP1 prior to stimulation of cells with ligand (Figure 5H). Additional analysis using PAE cells null for chimeric VEGFR-2 revealed that indeed association of SH2 domain of Src with IQGAP1 is independent of VEGFR-2 (data not shown). In contrast to the SH2 domain, the SH3 domain of c-Src failed to interact with IQGAP1 with or without stimulation of VEGFR-2 (Figure 5I). The data suggest that SH2 domain mediates the interaction of c-Src with IQGAP1 by a novel mechanism that is independent of tyrosine phosphorylation of IQGAP1.

### IQGAP1 signaling pathway is required for VEGF-induced angiogenesis

To establish the biological importance of IQGAP1 in VEGFR-2 signaling, we initially examined its role in proliferation of endothelial cells. To this end, we employed siRNA-mediated knockdown strategy and analyzed proliferation of endothelial cells in response to VEGF stimulation. IQGAP1 siRNA significantly reduced expression of IQGAP1 in HMVE cells (Figure 5L). We further analyzed these cells for their ability to undergo proliferation in response to VEGF. Silencing the expression of IQGAP1 in primary endothelial cells, but not control siRNA, significantly inhibited VEGF-dependent cell proliferation (Figure 5M), suggesting that VEGFR-2/c-Src orchestrated tyrosine phosphorylation of IQGAP1 serves an important role in endothelial cell proliferation. Our recent study indicates that IQGAP1 is capable of modulating activity of b-Raf [27], to test whether IQGAP1 activity is also required for b-Raf phosphorylation in endothelial cells in response to VEGFR-2 activation we tested phosphorylation of b-Raf where expression of IQGAP1 is knocked down by siRNA. The data show that stimulation of endothelial cells with VEGF promotes phosphorylation of b-Raf at Ser 455 and silencing expression of IQGAP1 notably reduced its phosphorylation (Figure 5N). Quantification of phosphorylation of Ser 455 of b-Raf based on the three independent experiments also is shown (Figure 5Q). The reduced phosphorylation of b-Raf in IQGAP1 siRNA treated cells was not due to the differential amount of protein since relatively equal amount of b-Raf protein is present in each group (Figure 5O). The expression of IQGAP1 in cells treated with control siRNA or IQGAP1 siRNA also is shown (Figure 5P). In short, the data indicate that VEGFR-2-dependent endothelial cell proliferation, in part, is established by Src, IQGAP1and b-Raf axis (Figure 5R).

**Figure 6:**
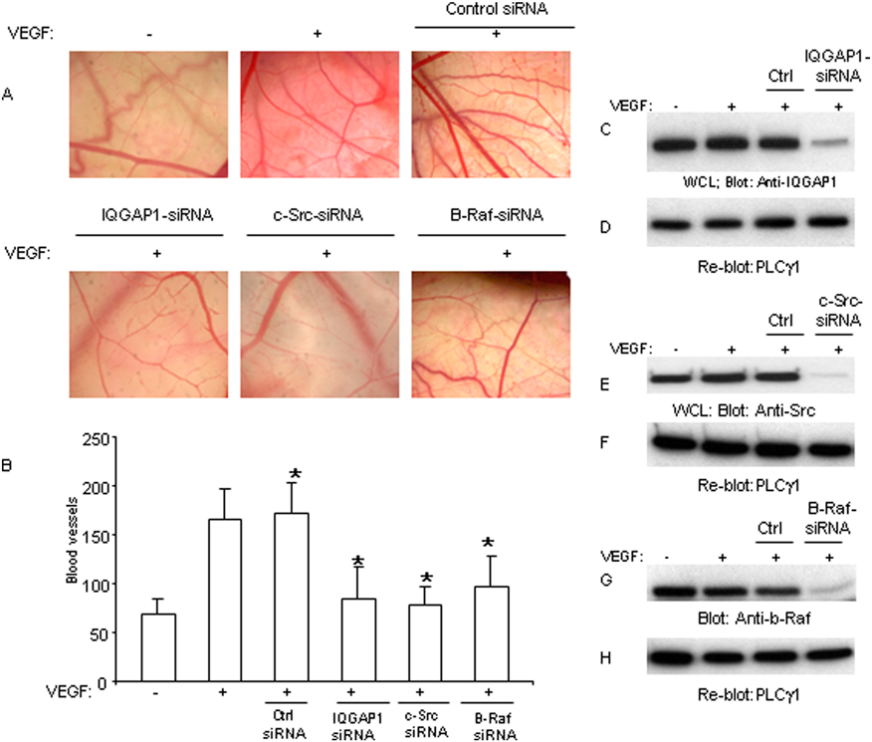
IQGAP1, Src and b-Raf activity is required for angiogenesis in vivo: 9-day old chicken chorioallantoic membrane (CAM) (n =  10 CAM for each experimental group) were prepared as described in Materials and Method section and exposed to filter paper disks saturated with siRNAs of either IQGAP1, Src, b-Raf or scrambled control siRNA for 24 hours and then incubated in the presence or in absence VEGF for an additional 72 hours. Pictures were taken by Leica Camera under microscope and a representative pictures from each group are shown (A). Blood vessels were quantified by Leica imaging software system. Each bar represents the mean ±SD of 10 CAM (n = 10). *P<0.05 versus control siRNA plus VEGF (B). CAM tissue was prepared and subjected to Western blot analysis using an anti-IQGAP1 antibody (C), anti-c-Src antibody (E), and anti-b-Raf antibody (G). The same membranes were re-blotted with an anti-PLCγ1 antibody as a control for protein loading (D, F, H).

To further address the physiological importance of c-Src, IQGAP1and b-Raf in angiogenesis in an *in vivo* setting, we used chicken chorioallantoic membrane (CAM) angiogenesis assay where their expression were knocked down by siRNA strategy. The result showed that targeting IQGAP1, c-Src and b-Raf individually by siRNA suppresses the ability of VEGF to stimulate angiogenesis (Figure 6A), where control siRNA had no negative effect on angiogenesis (Figure 6A). Furthermore, quantification of CAM assay showed that VEGF treatment of CAM induced robust angiogenic response where IQGAP1-siRNA, c-Src-siRNA and b-Raf-siRNA each decreased VEGF-induced angiogenesis (Figure 6B). As noted the siRNA-mediated inhibition of VEGF-induced angiogenesis was near to baseline angiogenesis (Figure 6B) underscoring the importance of this pathway for VEGF-induced angiogenesis. The ability of these specific siRNAs to knockdown expression of c-Src, IQGAP1 and b-Raf also are analyzed (Figure 6C, 6E, 6G). The same membranes were re-probed for PLCγ1 as a loading control (Figure 6D, 6F, 6H). In sum, our data demonstrate that IQGAP1, c-Src and b-Raf pathway plays a critical role both *in vitro* and *in vivo* in VEGF-mediated angiogenesis.

## Discussion

In this report, we have identified a novel angiogenic pathway emanating from phospho-Y1057 of VEGFR-2. Phosphorylation of Y1057 plays a combinatorial role in VEGFR-2 signaling; it recruits c-Src kinase to VEGFR-2, regulates phosphorylation of multi-docking Y1173 site, mediates VEGFR-2-dependent proliferation of endothelial cells and angiogenesis through IQGAP1 and b-Raf. One of the most interesting aspects of this study is the discovery of an unexpected role of Src family kinases in the phosphorylation of Y1173 of VEGFR-2. Y1173 of VEGFR-2 is considered to be a *bona fide* mediator of angiogenic signaling of VEGFR-2 *in vivo* since its mutation abrogates its ability to stimulate angiogenesis during mouse embryonic development [6], [11] and Y1173 functions as a multi-docking residue on VEGFR-2 that recruits p85/PI3kinase, PLCγ1, Shb, and Sck [3]–[7], [10]. Thus, phosphorylation of Y1173 by Src kinases represents one pathway by which the intensity and extent of Y1173-dependent signaling of VEGFR-2 is controlled. Conversely, inhibition of Src kinases could skew the binding of these signaling proteins to VEGFR-2 and their subsequent activation by VEGFR-2.

A recent study indicates that c-Cbl directly interacts with Y1057 of VEGFR-2 [14]. Interestingly, not only does c-Src bind directly to Y1057, but c-Cbl provides an additional mechanism for c-Src to interact with VEGFR-2 by serving as an “adaptor” which in turn may modulate its activation by VEGFR-2. Activation of c-Src is linked to vascular permeability [16], endothelial cell survival and angiogenesis [16], [17]. Our findings show that although c-Src activity is dispensable for VEGFR-2-dependent tube formation of endothelial cells, its activity is highly critical for VEGF-stimulated endothelial cell proliferation and angiogenesis in CAM model of angiogenesis. The mechanism by which c-Src contributes to VEGFR-2-dependent cell proliferation is linked, in part to its ability to phosphorylate Y1173, which is known to regulate VEGFR-2-mediated endothelial cell proliferation [5]–[7]. c-Src also associates with IQGAP1 in endothelial cells and catalyzes tyrosine phosphorylation of IQGAP1 which acts as a downstream target of VEGFR-2 and c-Src to stimulate endothelial cell proliferation. A recent work has demonstrated that c-Src regulates activation of Raf-1 (also known as c-Raf) in endothelial cells [17]. c-Src also associates directly with another Raf family protein, b-Raf, leading to activation of MAPK pathway in a Ras-independent manner [27], [33]. Our demonstration that IQGAP1 is activated by VEGFR-2 further supports earlier studies that shows VEGFR-2 activates MAPK pathway in Ras-independent manner [31].

In conclusion, in this study we have identified and defined a molecular switch by which VEGFR-2 regulates endothelial cell proliferation and stimulates angiogenesis by identifying Y1057 of VEGFR-2 that undergoes phosphorylation and orchestrates recruitment and activation of c-Src and modulates endothelial cell proliferation via IQGAP1-dependent signaling pathway.

## Materials and Methods

### Reagents, antibodies, siRNAs and vectors

Recombinant human VEGF-A and colony stimulating factor-1 (CSF-1) were purchased from R&D Systems (Minneapolis, MN). Mouse monoclonal anti-phosphotyrosine antibody 4G10 and anti-IQGAP1 antibody were purchased from Upstate Biotechnology (Lake Placid, NY). Rabbit anti-phospho-VEGFR-2 (pY1052) and rabbit polyclonal anti-phospho-VEGFR-2 (pY1057) were made in collaboration with Upstate Biotechnology (Lake Placid, NY). Rabbit polyclonal anti-VEGFR-2 sera were raised against either a glutathione S-transferase-VEGFR-2 kinase insert domain fusion protein (1410) or a GST-VEGFR-2 carboxyl-terminus fusion protein (1412) [18], [21]. Rabbit monoclonal anti-phospho-VEGFR-2 (pY1173) was purchased from Cell Signaling Technology (Beverly, MA). Rabbit polyclonal anti-PLCγ1 was purchased from Santa Cruz Biotechnology (Santa Cruz, CA). The oligonucleotide inserted into pSUPER.retro.puro vector used to produce c-Cbl siRNA was described elsewhere [14], and other oligonucleotide siRNA used in this study are described in supplementary Data S1.

### Cell culture, cell lines and retroviral infection

Porcine aortic endothelial (PAE) cells were grown in 10% FBS cells and lack endogenous expression of VEGFR-2 [20]. Primary human dermal microvascular endothelial cells (HMVE cells) were purchased from (Cell Applications, Inc. San Diego) and human umbilical vein endothelial (HUVE) cells, bovine vascular endothelial cells (BAE) cells and human embryonic kidney cells (HEK-293) cells were purchased from ATCC and were grown in 10% FBS plus growth factor supplements and penicillin/ streptomycin. SYF cells are triple knockout (Src, Yes and Fyn) fibroblast cells and they were used as described [15]. Creation of the VEGFR-2 chimera (CKR), in which the extracellular domain of VEGFR-2 is replaced with that of the human CSF-1R, has been previously described [18]. The oligonucleotide siRNA for c-Src was 5′-AAACTCCCCTTG-CTCATGTAC-3′. The oligonucleotide siRNA used for IQGAP1was cloned into pSUPER.retro.puro vector as previously described [27]. Avian siRNAs generated for c-Src, IQGAP1 and b-Raf are the following; siRNAs of avian c-Src were; CAUUGCCAAGGUCAGCGAU UU and AUGACGCCAC AGCGCAGGC UU, siRNA for avian b-raf were; UUGGCUGGGACACUGACAU UU and AGGAUAGGAUCUGGAUCAU UU; siRNA for avian IQGAP1 was; ACUCUGCAAGCCUUACA GAUU. Creation of mutant VEGFR-2s and other related methods are described in supplementary Data S1.

### *In vitro* angiogenesis/tubulogenesis assay

Angiogenesis assay was performed essentially as described [14], [28]. Briefly, PAE cells were suspended in DMEM containing 1% fetal bovine serum and 0.24% (w/v) carboxymethylcellulose (4000 centipoise) in non-adherent round-bottom 96-well plates. After 24 h, all cells formed one single spheroid per well. Spheroids were cultured for additional 48 h before using them in the in vitro angiogenesis assay in the manner previously described [14], [28]. Sprouting and tubulogenesis was observed under an inverted phase-contrast microscope and pictures were taken using a Leica digitial camera system. For statistical analysis purposes four sprouting colonies were used for each experimental condition and their sprouting was quantified using image J program (NIH).

### Immunoprecipitation, immunoblotting and *in vitro* pull-down assay

Cells were prepared for immunoprecipitation as described [14]. Briefly, cells were grown in 10-cm culture dishes until 80–90% confluent and after serum starvation, cells were left either resting or stimulated with appropriate ligands as indicated in figure legends. Cells were lysed and normalized whole cell lysates were subject to immunoprecipitation by incubating with appropriate antibodies. Immunocomplexes were captured by incubating with either Protein-A Sepharose or Protein-G-Agarose beads and immunoprecipitated protein were subjected to immunoblotting analysis. Additional experimental details are provided in Supplementary Data S1. In some occasions the blots were scanned and they were subsequently quantified using *Image J* program (NIH).

### Statistical Analysis

For statistical purposes the blots of three independent experiments were scanned and blots were quantified using NIH Image J program. Comparison of the different parameters for the blots was determined by repeated measures analysis of variance (ANOVA). Significant differences were assigned using Kruskal-Wallis post hoc test. The criterion for significance for all tests was set at p < 0.05. Specific software was used to assist in the data analysis (GraphPadPrism v4.0b, GraphPad Software, San Diego, USA).

### GST-Pull down assay

*In vitro* GST fusion protein binding experiments were performed as described [30]. To this end, equal numbers of endothelial cells were grown to 90% confluency prior to serum starvation for overnight. Unstimulated or ligand-stimulated cells were lysed in ice-cold lysis buffer supplemented with 2 mM Na_3_VO_4_ and a protease inhibitor cocktail. Equal amounts of the appropriate immobilized GST fusion proteins were incubated with normalized whole cell lysates by rocking for 3 h at 4°C. The beads were washed four times in the presence of protease inhibitors and Na_3_VO4, and proteins were eluted and analyzed in Western blot analysis using appropriate antibody.

### Dot blot assay

Purified recombinant GST, GST-SH2-c-Src and GST-SH3-c-Src were eluted from glutathione-Sepharose beads and subjected to dot blot. Further information regarding this assay is provided in Supplementary Data S1.

### Cell proliferation assay

Cell proliferation was evaluated by direct cell counting. Endothelial cells were seeded at a density of 2×10^4^ cells/well in 24-well plates and cultured overnight; the cells were then incubated in serum-free medium for 12 hours. Cells were stimulated with recombinant human CSF-1 or VEGF-A at different concentrations as indicated in figure legends, and after 48 hours they were washed with PBS, harvested by mild trypsinization, and counted with a hematocytometer. Experiments were performed in triplicate and 3 separate experiments were performed and where appropriate, values were presented as means of ± SD.

### Chicken chorioallantoic membrane (CAM) angiogenesis assay

Pathogen free grade fertilized chick embryos (purchased from SPAFAS, Preston, CT) were incubated for 10 days (37°C with 70% humidity). A hole was made with a drill over the air sac at the end of the egg as described [16]. A small window was cut in the egg shell over the dropped CAM, in order to place growth factor and siRNA on the CAM [16], [17]. Filter disks were soaked in cortisone acetate, VEGF (200 ηg), siRNA and plus transfection agent and the disk filters were applied to the CAM of 9 day chick embryos and incubated at 37°C for 72 hours. 10 CAM were used per each group. Pictures were taken by Leica Camera under microscope from three different area randomly and these areas were further used to quantify blood vessels using Leica imaging software system. Statistical Analysis: Data represent mean ±SD of 10 CAM which includes three different fields from each CAM was used unless otherwise stated. Statistical significance was calculated by ANOVA soft ware program (prism v4.0b), considering p<0.05 as statistically significant. Tissue preparation for Western blotting: CAM tissue was also harvested for Western blotting. For this purpose CAM tissue was snap frozen in liquid nitrogen and samples were homogenized in a modified RIPA buffer. Five CAMs from each experimental group were homogenized in 1 ml of the RIPA buffer. Equal amounts of protein were subjected to Western blot analysis using desired antibody as described in the figure legends.

## Supporting Information

Data S1 (0.03 MB DOC)

Figure S1 Effect of mutation of tyrosines 1052 and 1057 to phenylalanine: Equal number of serum-starved PAE cells expressing wild type CKR, F1052/CKR, F1057/CKR and F1052/F1057/CKR were either unstimulated (−) or stimulated with CSF-1 (+) for 10 minutes. Total cell lysates were subjected to Western blot analysis using an anti-phosphoY1052 specific-VEGFR-2 antibody (A), an anti-phosphoY1057 specific anti-VEGFR-2 antibody (B), an anti-phosphotyrosine antibody (C), and an anti-VEGFR-2 antibody (D). Equal number of serum-starved PAE cells expressing wild type CKR, F1052/CKR, F1057/CKR and F1052/F1057/CKR without stimulation were lysed and immunoprecipitated with an anti-VEGFR-2 antibody. The immunoprecipitated proteins were subjected to an in vitro kinase assay with increasing concentrations of ATP as indicated and described in Materials and Methods and immunoblotted with an anti-phosphotyrosine antibody (E). The same membranes were re-blotted with an anti-VEGFR-2 antibody for proteins levels (F).
(0.22 MB TIF)

Figure S2 Effect of mutation of Tyrosines 1052 and 1057 to glutamic acid. Partial amino acid sequence of mouse VEGFR-2 containing Y1052 and Y1057 and the schematic presentation of VEGFR-2 are shown (A). Equal number of serum-starved PAE cells expressing wild type CKR, E1052/CKR, E1057/CKR and E1052/E1057/CKR were either unstimulated (−) or stimulated with CSF-1 (+) for 10 minutes. Total cell lysates were subjected to Western blot analysis using an anti-phosphoY1052 specific-VEGFR-2 antibody (B), an anti-phosphoY1057 specific anti-VEGFR-2 antibody (C), an anti-phosphotyrosine antibody (D), and an anti-VEGFR-2 antibody (E). Equal number of serum-starved PAE cells expressing wild type CKR, E1052/CKR, E1057/CKR and E1052/E1057/CKR without stimulation were lysed and immunoprecipitated with an anti-VEGFR-2 antibody. The immunoprecipitated proteins were subjected to an in vitro kinase assay with increasing concentrations of ATP as indicated and described in Materials and Methods and immunoblotted with an anti-phosphotyrosine antibody (F). The same membranes were re-blotted with an anti-VEGFR-2 antibody for proteins levels (G). The image analysis program was used to quantify the data. The tyrosine phosphorylation values were normalized over total protein (H). Arbitrary unit (AU).
(0.24 MB TIF)

Figure S3 GST-Src but not GST binds to VEGFR-2: Serum-starved PAE cells expressing CKR were either unstimulated (−) or stimulated (+) with CSF-1 for 10 minutes, lysed, and whole cell lysates were incubated with purified GST alone or GST-SH2-Src protein. The GST-SH2-Src bound proteins were subjected to Western blot analysis using an anti-VEGFR-2 antibody (A). Whole cell lysates were blotted with anti-VEGFR-2 antibody as a control (B).
(0.09 MB TIF)
