# Supplementary material for: IQGAP1-Dependent Signaling Pathway Regulates Endothelial Cell Proliferation and Angiogenesis
Source: PLoS One. 2008 Dec 3;3(12):e3848. doi: 10.1371/journal.pone.0003848 (PMC2585478; doi:10.1371/journal.pone.0003848)
Supplement: Data S1 — (0.03 MB DOC) [file pone.0003848.s001.doc]

**Supplementary Data**

**Results**:

**Tyrosines 1052 and 1057 of VEGFR-2 are phosphorylated *in vivo***

Tyrosines 1052 and 1057 are located in the sensitive kinase domain of VEGFR-2 commonly known as activation loop (23). To determine whether these tyrosines are phosphorylated *in vivo* we have developed two phospho-specific antibodies against Y1052 and Y1057. The results show that tyrosines 1052 and 1057 are phosphorylated in a ligand-dependent manner in PAE cells (**Figure S2B, S2C**). Mutation of Y1052 and Y1057 to glutamic acid (E) eliminates the ability of these phospho-specific antibodies to recognize these sites, respectively (**Figure S2B, S2C**). Moreover, the data show that mutation of either Y1052 or Y1057 also has no effect on the phosphorylation of each other and their double mutation totally abolishes the ability of these antibodies to recognize these sites (**Figure S2B, S2C**). To further analyze the contribution of Y1052 and Y1057 to the kinase activation of VEGFR-2, we analyzed the effect of single and double mutation of Y1052 and Y1057 in the kinase activation of VEGFR-2 in an *in vitro* kinase assay. Mutation of either Y1052 or Y1057 slightly (~20%) reduced kinase activation of VEGFR-2 in the presence of 1mM ATP (**Figure S2F and S2H**). In contrast, double mutation of Y1052 and Y1057 yielded a VEGFR-2 that failed to undergo tyrosine phosphorylation (**Figure S2F and S2H**). Altogether, these data show that Y1052 and Y1057 are phosphorylated *in vivo* and single mutation of either Y1052 or Y1057 is dispensable for kinase activation of VEGFR-2. However, phosphorylation of both Y1052 and Y1057 are essential for full kinase activation of VEGFR-2. Moreover, data show that phosphorylation of Y1052 and Y1057, in particular, Y1057 plays a dual role in VEGFR-2 function; it directly participates in the recruitment of signaling proteins to VEGFR-2 and along with Y1052 modulates kinase activation of VEGFR-2.

**Figure S1: Effect of mutation of tyrosines 1052 and 1057 to phenylalanine:** Equal number of serum-starved PAE cells expressing wild type CKR, F1052/CKR, F1057/CKR and F1052/F1057/CKR were either unstimulated (-) or stimulated with CSF-1 (+) for 10 minutes. Total cell lysates were subjected to Western blot analysis using an anti-phosphoY1052 specific-VEGFR-2 antibody (**A**), an anti-phosphoY1057 specific anti-VEGFR-2 antibody (**B**), an anti-phosphotyrosine antibody (**C**), and an anti-VEGFR-2 antibody (**D**). Equal number of serum-starved PAE cells expressing wild type CKR, F1052/CKR, F1057/CKR and F1052/F1057/CKR without stimulation were lysed and immunoprecipitated with an anti-VEGFR-2 antibody. The immunoprecipitated proteins were subjected to an in vitro kinase assay with increasing concentrations of ATP as indicated and described in Materials and Methods and immunoblotted with an anti-phosphotyrosine antibody (**E**). The same membranes were re-blotted with an anti-VEGFR-2 antibody for proteins levels (**F**).

**Figure S2: Effect of mutation of Tyrosines 1052 and 1057 to glutamic acid.** Partial amino acid sequence of mouse VEGFR-2 containing Y1052 and Y1057 and the schematic presentation of VEGFR-2 are shown (**A**). Equal number of serum-starved PAE cells expressing wild type CKR, E1052/CKR, E1057/CKR and E1052/E1057/CKR were either unstimulated (-) or stimulated with CSF-1 (+) for 10 minutes. Total cell lysates were subjected to Western blot analysis using an anti-phosphoY1052 specific-VEGFR-2 antibody (**B**), an anti-phosphoY1057 specific anti-VEGFR-2 antibody (**C**), an anti-phosphotyrosine antibody (**D**), and an anti-VEGFR-2 antibody (**E**). Equal number of serum-starved PAE cells expressing wild type CKR, E1052/CKR, E1057/CKR and E1052/E1057/CKR without stimulation were lysed and immunoprecipitated with an anti-VEGFR-2 antibody. The immunoprecipitated proteins were subjected to an in vitro kinase assay with increasing concentrations of ATP as indicated and described in Materials and Methods and immunoblotted with an anti-phosphotyrosine antibody (**F**). The same membranes were re-blotted with an anti-VEGFR-2 antibody for proteins levels (**G**). The image analysis program was used to quantify the data. The tyrosine phosphorylation values were normalized over total protein (**H**). Arbitrary unit (AU).

**Figure S3:** **GST-Src but not GST binds to VEGFR-2**: Serum-starved PAE cells expressing CKR were either unstimulated (-) or stimulated (+) with CSF-1 for 10 minutes, lysed, and whole cell lysates were incubated with purified GST alone or GST-SH2-Src protein. The GST-SH2-Src bound proteins were subjected to Western blot analysis using an anti-VEGFR-2 antibody (A). Whole cell lysates were blotted with anti-VEGFR-2 antibody as a control (**B**).

**Materials Methods**:

**Cell culture**: Retroviral expression of wild type VEGFR-2, CKR and tyrosine mutant CKRs: F936/CKR, F949/CKR, F994/CKR, F1052/CKR, F1057/CKR, F1173/CKR and C-tail truncated CKR in porcine aortic endothelial (PAE) cells have been previously described (14,18,28,29). Persistent knockdown of endogenous IQGAP1 and c-Cbl expression in endothelial cells was achieved through retroviral transduction using the pSUPER.retro.puro vector (OligoEngine, Seattle, WA). Retroviral expression plasmids were transiently transfected into the 293-GPG retroviral packaging cell line using LipofectAMINE (Invitrogen, Carlsbad, CA). After 72 h, supernatants were collected and used to transduce endothelial cells. Cells were selected with the appropriate antibiotics (G418; 2 mg/ml or Puromycin; 4 μg/ml) and used as pooled populations for experiments. Some experiments were performed in HEK-293 cells using standard method of transient transfection system and all the constructs were cloned into pcDNA3.1 vector.
