# Supplementary figures and images for: IQGAP1-Dependent Signaling Pathway Regulates Endothelial Cell Proliferation and Angiogenesis
Source: PLoS One. 2008 Dec 3;3(12):e3848. doi: 10.1371/journal.pone.0003848 (PMC2585478; doi:10.1371/journal.pone.0003848)

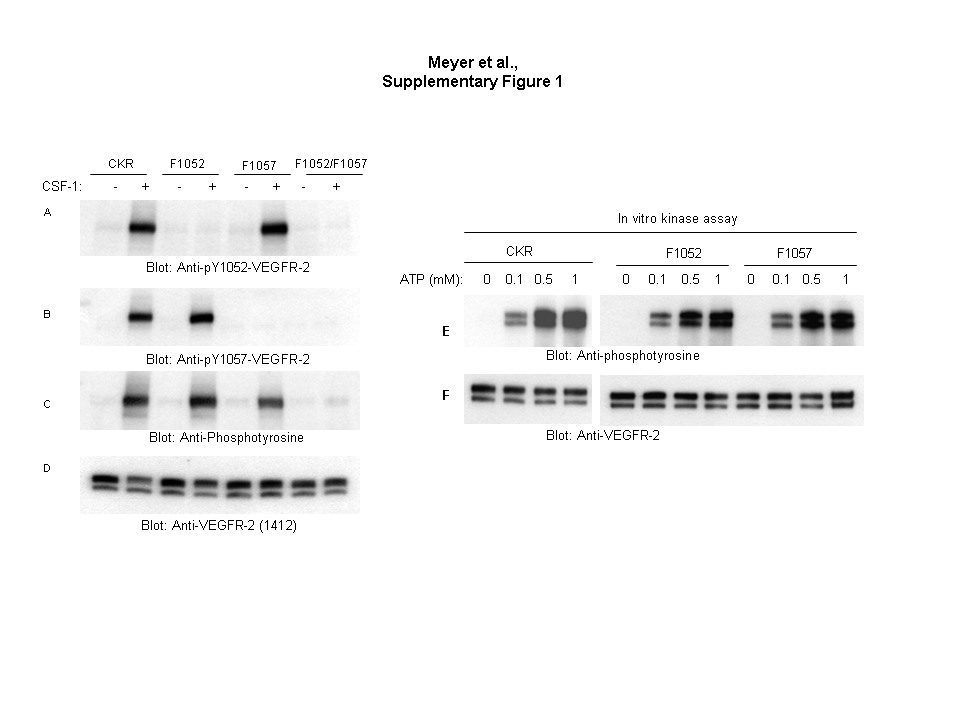

Supplement: Figure S1 — Effect of mutation of tyrosines 1052 and 1057 to phenylalanine: Equal number of serum-starved PAE cells expressing wild type CKR, F1052/CKR, F1057/CKR and F1052/F1057/CKR were either unstimulated (−) or stimulated with CSF-1 (+) for 10 minutes. Total cell lysates were subjected to Western blot analysis using an anti-phosphoY1052 specific-VEGFR-2 antibody (A), an anti-phosphoY1057 specific anti-VEGFR-2 antibody (B), an anti-phosphotyrosine antibody (C), and an anti-VEGFR-2 antibody (D). Equal number of serum-starved PAE cells expressing wild type CKR, F1052/CKR, F1057/CKR and F1052/F1057/CKR without stimulation were lysed and immunoprecipitated with an anti-VEGFR-2 antibody. The immunoprecipitated proteins were subjected to an in vitro kinase assay with increasing concentrations of ATP as indicated and described in Materials and Methods and immunoblotted with an anti-phosphotyrosine antibody (E). The same membranes were re-blotted with an anti-VEGFR-2 antibody for proteins levels (F). (0.22 MB TIF) [file pone.0003848.s002.tif]

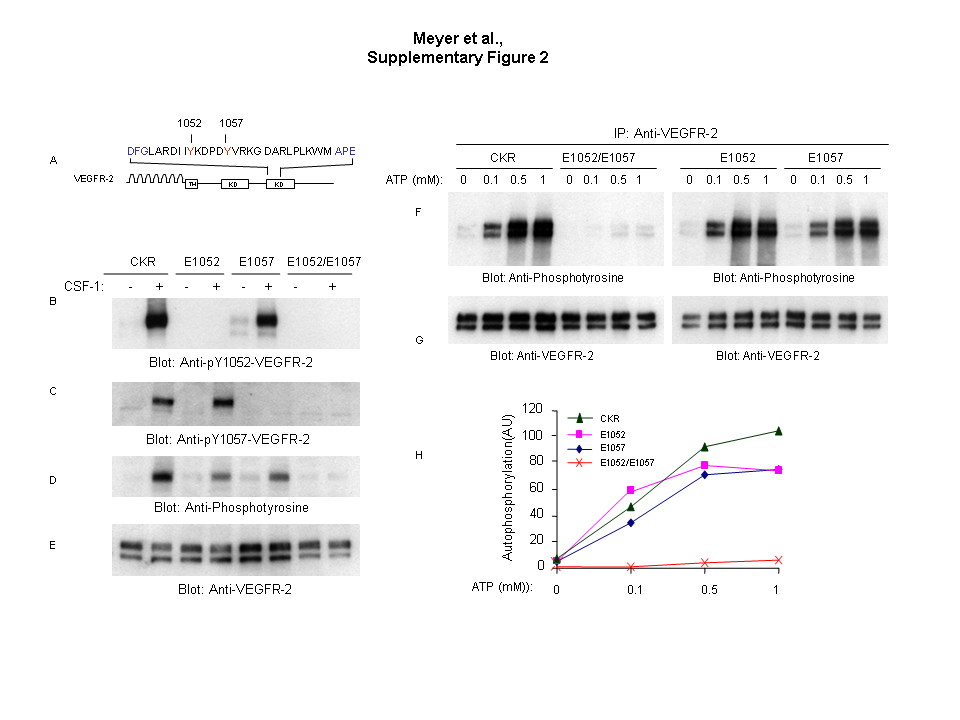

Supplement: Figure S2 — Effect of mutation of Tyrosines 1052 and 1057 to glutamic acid. Partial amino acid sequence of mouse VEGFR-2 containing Y1052 and Y1057 and the schematic presentation of VEGFR-2 are shown (A). Equal number of serum-starved PAE cells expressing wild type CKR, E1052/CKR, E1057/CKR and E1052/E1057/CKR were either unstimulated (−) or stimulated with CSF-1 (+) for 10 minutes. Total cell lysates were subjected to Western blot analysis using an anti-phosphoY1052 specific-VEGFR-2 antibody (B), an anti-phosphoY1057 specific anti-VEGFR-2 antibody (C), an anti-phosphotyrosine antibody (D), and an anti-VEGFR-2 antibody (E). Equal number of serum-starved PAE cells expressing wild type CKR, E1052/CKR, E1057/CKR and E1052/E1057/CKR without stimulation were lysed and immunoprecipitated with an anti-VEGFR-2 antibody. The immunoprecipitated proteins were subjected to an in vitro kinase assay with increasing concentrations of ATP as indicated and described in Materials and Methods and immunoblotted with an anti-phosphotyrosine antibody (F). The same membranes were re-blotted with an anti-VEGFR-2 antibody for proteins levels (G). The image analysis program was used to quantify the data. The tyrosine phosphorylation values were normalized over total protein (H). Arbitrary unit (AU). (0.24 MB TIF) [file pone.0003848.s003.tif]

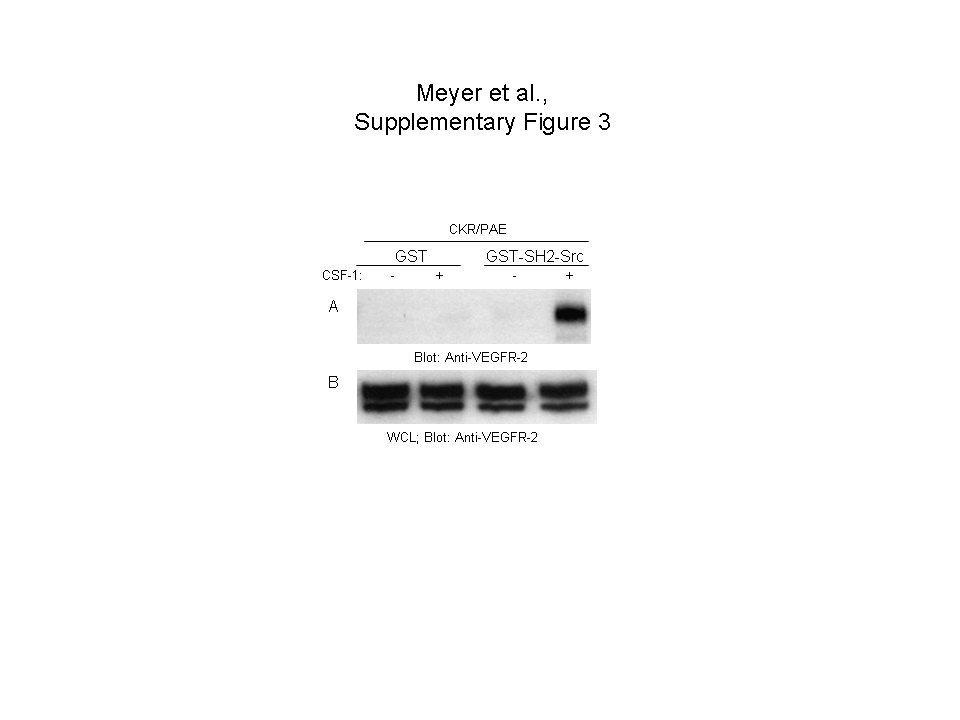

Supplement: Figure S3 — GST-Src but not GST binds to VEGFR-2: Serum-starved PAE cells expressing CKR were either unstimulated (−) or stimulated (+) with CSF-1 for 10 minutes, lysed, and whole cell lysates were incubated with purified GST alone or GST-SH2-Src protein. The GST-SH2-Src bound proteins were subjected to Western blot analysis using an anti-VEGFR-2 antibody (A). Whole cell lysates were blotted with anti-VEGFR-2 antibody as a control (B). (0.09 MB TIF) [file pone.0003848.s004.tif]
